# Alterations in microbiota of patients with COVID-19: potential mechanisms and therapeutic interventions

**DOI:** 10.1038/s41392-022-00986-0

**Published:** 2022-04-29

**Authors:** Bin Wang, Lei Zhang, Yongqiang Wang, Tong Dai, Ziran Qin, Fangfang Zhou, Long Zhang

**Affiliations:** 1 MOE Laboratory of Biosystems Homeostasis & Protection and Innovation Center for Cell Signaling Network, Life Sciences Institute, Zhejiang University, 310058 Hangzhou, PR China; 2 Department of Orthopaedic Surgery, The First Affiliated Hospital of Wenzhou Medical University, 325000 Wenzhou, PR China; 3 Institutes of Biology and Medical Science, Soochow University, 325200 Suzhou, PR China

**Keywords:** Microbiology, Infectious diseases

## Abstract

The global coronavirus disease 2019 (COVID-19) pandemic is currently ongoing. It is caused by severe acute respiratory syndrome coronavirus 2 (SARS-CoV-2). A high proportion of COVID-19 patients exhibit gastrointestinal manifestations such as diarrhea, nausea, or vomiting. Moreover, the respiratory and gastrointestinal tracts are the primary habitats of human microbiota and targets for SARS-CoV-2 infection as they express angiotensin-converting enzyme-2 (ACE2) and transmembrane protease serine 2 (TMPRSS2) at high levels. There is accumulating evidence that the microbiota are significantly altered in patients with COVID-19 and post-acute COVID-19 syndrome (PACS). Microbiota are powerful immunomodulatory factors in various human diseases, such as diabetes, obesity, cancers, ulcerative colitis, Crohn’s disease, and certain viral infections. In the present review, we explore the associations between host microbiota and COVID-19 in terms of their clinical relevance. Microbiota-derived metabolites or components are the main mediators of microbiota-host interactions that influence host immunity. Hence, we discuss the potential mechanisms by which microbiota-derived metabolites or components modulate the host immune responses to SARS-CoV-2 infection. Finally, we review and discuss a variety of possible microbiota-based prophylaxes and therapies for COVID-19 and PACS, including fecal microbiota transplantation (FMT), probiotics, prebiotics, microbiota-derived metabolites, and engineered symbiotic bacteria. This treatment strategy could modulate host microbiota and mitigate virus-induced inflammation.

## Introduction

There are tenfold more bacterial cells in the human microbiota than there are human tissue cells and there are 100-fold more bacterial than human genes.^1–4^ These bacteria inhabit all surfaces of the human body including the gastrointestinal and respiratory tracts.^5–8^ The human body selectively permits certain bacteria to colonize it, and it furnishes them with a suitable habitat. Microbiota serve multiple important functions in and on the human body such the decomposition of indigestible carbohydrates and proteins, nutrient digestion and absorption, vitamin biosynthesis, and host immunity induction, instruction, and function.^9–13^ The microbiota influence human health and are associated with several diseases.

**Fig. 1:**
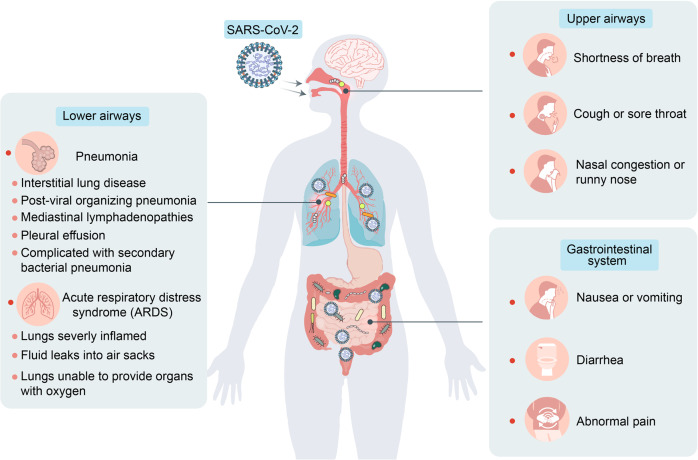
COVID-19-associated respiratory and gastrointestinal symptoms. Various respiratory and gastrointestinal manifestations occur in patients with COVID-19 including shortness of breath, cough or sore throat, nasal congestion or runny nose, pneumonia, acute respiratory distress syndrome (ARDS), nausea or vomiting, diarrhea, and abdominal pain

The global coronavirus disease 2019 (COVID-19) pandemic is caused by severe acute respiratory syndrome coronavirus 2 (SARS-CoV-2) and has posed serious threats to public health and the global economy.^14^ Patients with COVID-19 present with symptoms of respiratory infection including fever, fatigue, abnormal chest X-ray, cough, and shortness of breath.^15–18^ Furthermore, a high proportion of COVID-19 patients also exhibit gastrointestinal manifestations such as diarrhea, nausea or vomiting, anorexia, and abdominal pain (Fig. 1).^19–21^ Evidence from clinical studies suggests that respiratory and gastrointestinal microbiota homeostasis is disrupted in hospitalized COVID-19 patients.^22–27^ SARS-CoV-2 may predispose patients to secondary pathogen infections of the respiratory and gastrointestinal tracts. These are responsible for much of the morbidity and mortality associated with COVID-19.^28,29^ Therefore, microbiota may play important roles in SARS-CoV-2 infection.

The aim of this review was to summarize the relationships between microbiota and COVID-19 in terms of their clinical relevance and immunological mechanisms. We also explored various interventions that target microbiota, are based on the immunological interplay between the microbiota and COVID-19, and could optimize anti-SARS-CoV-2 therapies.

## Microbiota and COVID-19

### Respiratory and gastrointestinal tracts are primary habitats of human microbiota and targets for SARS-CoV-2 infection

**Fig. 2:**
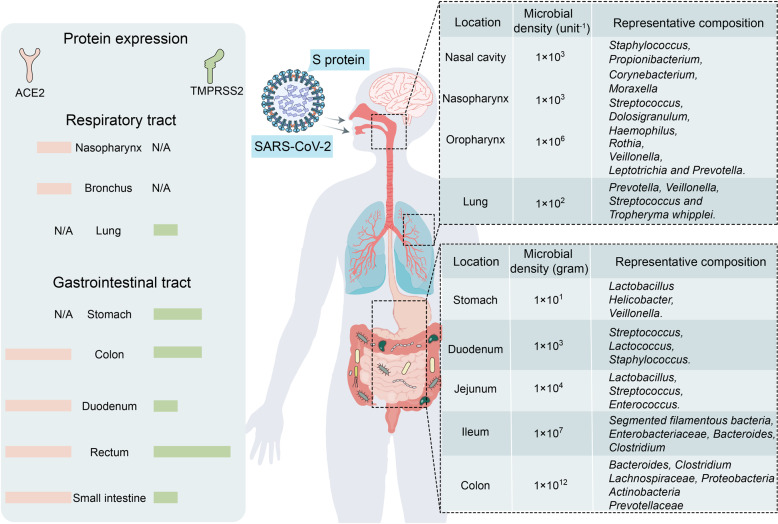
Primary habitats of human microbiota: respiratory and gastrointestinal tracts as SARS-CoV-2 infection targets. SARS-CoV-2 receptors ACE2 and TMPRSS2 are expressed mainly in respiratory and gastrointestinal tracts which provide many suitable habitats for microorganisms. The right side of the figure lists representative bacterial populations in different parts of the respiratory and gastrointestinal tracts

SARS-CoV-2 is the causative agent of COVID-19. It is a single-stranded, positive-sense RNA virus of the genus *Betacoronavirus.*^30,31^ It encodes membrane (M), nucleocapsid (N), spike (S), and envelope (E) structural proteins and multiple non-structural proteins.^32^ SARS-CoV-2 obligately requires the S protein to penetrate host cells.^33^ On the virion, the S protein is a homotrimer comprising S1 and S2 subunits. The former binds host angiotensin-converting enzyme-2 (ACE2) while the latter mediates membrane fusion.^34–36^ The virus hijacks host cell-surface proteases such as transmembrane protease serine 2 (TMPRSS2) which, in turn, activates viral S protein, cleaves ACE2 receptors, and facilitates viral binding to the host cell membrane.^37–39^ In addition to ACE2 and TMPRSS2-mediated entry, SARS-Cov-2 can also utilize the phagocytosis or endocytosis function of host cells to invade certain immune cell types such as macrophages.^40^ ACE2 and TMPRSS2 are strongly expressed in the respiratory and gastrointestinal tracts. As the latter communicates with the external environment, they are the major targets of SARS-CoV-2 invasion (Fig. 2).^41–46^ Moreover, both of these organ systems harbor large microbial populations.

Gas exchange is the primary function of the respiratory tract. To perform gas exchange efficiently, adult human airways have approximately 40-fold larger surface area than skin.^47^ However, this tissue surface also provides numerous habitats suitable for microorganisms. High bacterial densities (103–106 U^−1^) occur in healthy upper airways including the nasal cavity, nasopharynx, and oropharynx. In contrast, the lung has slightly lower bacterial densities (~102 U^−1^).^48^ Healthy upper airways are typically populated by *Staphylococcus, Propionibacterium, Leptotrichia, Rothia, Dolosigranulum, Haemophilus, Moraxella, Veillonella*, and *Corynebacterium*. *Veillonella, Fusobacterium, and Haemophilus* are the main genera inhabiting healthy lungs. *Prevotella* and *Streptococcus* occur in both upper airways and lungs.^5,48–50^ Evidence from prior research demonstrated that commensal bacteria in the respiratory tract help prevent pathogens from establishing infections and spreading on the mucosal surfaces.^48,51^ This phenomenon is known as “colonization resistance”. Hence, respiratory tract microbiota might help prevent SARS-CoV-2 infection. By preventing SARS-CoV-2 colonization on the mucosal surfaces, microbiota could inhibit the virus infection to a certain degree.

Respiratory droplet and fomite transmission may be the primary modes of SARS-CoV-2 transmission. Nevertheless, a recent study suggested that SARS-CoV-2 may also be spread via the fecal–oral route.^52^ As ACE2 and TMPRSS2 are highly expressed in the gastrointestinal tract, SARS-CoV-2 also targets the gut.^45,46^ Several studies reported that stool samples from patients with COVID-19 were positive for SARS-CoV-2 viral RNA. Endoscopy revealed colon damage in these patients. Thus, SARS-CoV-2 can infect the gastrointestinal tract.^53–58^ A population-based study conducted in China showed that viral RNA was detected in the stool samples of ≤53% of all COVID-19 patients.^59^ A biopsy performed on a COVID-19 patient disclosed the SARS-CoV-2 protein coat in the stomach, duodenum, and rectum.^59^ Therefore, both SARS-CoV-2 and its close relative SARS-CoV can infect the gut. The gut microflora are more abundant and diverse than those in the respiratory tract.^60^ A few studies confirmed that gut microbiota help regulate intestinal immune homeostasis and pathogen infection.^61–63^ For this reason, gut bacteria may be vital to the host immune response to SARS-CoV-2 infection.

### Gastrointestinal and respiratory symptoms of COVID-19 link microbiota with SARS-CoV-2 infection

The disease course of COVID-19 is characterized by the incubation, symptomatic, hyperinflammation, and resolution periods.^64^ The incubation period is usually ~1–14 d. In most cases, though, it is 3–7 d.^16^ Approximately 97.5% of all COVID-19 patients develop symptoms within 14 d of infection. Only 2.5% of them remain asymptomatic.^16^ The clinical manifestations of COVID-19 are highly variable but commonly include shortness of breath (53–80%), sputum production (34.3%), dry cough (60–86%), and sore throat (13.9%).^19^

Several clinical studies reported that 11–39% of all COVID-19 patients have gastrointestinal symptoms, including nausea, vomiting, diarrhea, and abdominal pain (Fig. 1).^21,55,65–81^ A study conducted in Chile reported that out of 7,016 patients with COVID-19, 11% displayed gastrointestinal symptoms.^74^ Jin et al. reported that among 651 patients with COVID-19 in Zhejiang, China, 8.6% exhibited diarrhea while 4.15% presented with nausea or vomiting.^21^ Gastrointestinal symptoms are associated with a relatively higher risk of hospitalization and/or greater disease severity. In severe and/or critical patients, the disease progresses and causes complications such as acute respiratory distress syndrome (ARDS), sepsis, secondary pathogen pneumonia and end-stage organ failure. As microbiota maintain respiratory and gastrointestinal homeostasis and health, the foregoing COVID-19-associated symptoms may link microbiota with SARS-CoV-2 infection.

### Microbiota eubiosis is disturbed in patients with COVID-19

Emerging evidence suggests that the microbiota of the respiratory and gastrointestinal tracts are dramatically altered in COVID-19 patients. An early study in Guangdong Province, China revealed that the respiratory microbiota in COVID-19 patients have reduced α-diversity and elevated levels of opportunistic pathogenic bacteria.^82^ The researchers detected concomitant rhinovirus B, human herpes alphavirus 1 and human orthopneumovirus infection in 30.8% (4/13) of all severe COVID-19 patients but not in any mild cases. The major respiratory microbial taxa in the critically ill COVID-19 patients consisted of *Burkholderia cepacia complex (BCC)*, *Staphylococcus epidermidis*, and/or *Mycoplasma* spp. In 23.1% (3/13) of all severe COVID-19 cases, clinical sputum and/or nasal secretion cultures confirmed the presence of *BCC* and *S. epidermidis*. In a critical COVID-19 patient, there was a time-dependent secondary *Burkholderia cenocepacia* infection and expression of multiple virulence genes that might have accelerated disease progress and hastened eventual death. A study conducted at Huashan Hospital in Shanghai, China reported that among 62 COVID-19 and 125 non-COVID-19 pneumonia cases, potentially pathogenic microbes were detected in 47% of the former, and 58% of the pathogens were respiratory viruses.^24^ A recent study demonstrated a link between respiratory microbiota and COVID-19 disease severity.^23^ Several potential confounding factors contributed to microbiota alteration in COVID-19. These included time spent in the intensive care unit (ICU), antibiotic administration, and type of oxygen support. The authors integrated microbiome sequencing, viral load determination, and immunoprofiling, and identified specific oral bacteria associated with relatively higher levels of proinflammatory markers in COVID-19 patients.

Gut dysbiosis in COVID-19 patients was also investigated. A shotgun metagenomics analysis of 15 COVID-19 patients hospitalized in Hong Kong disclosed that their fecal microbiomes were deficient in beneficial commensals and abundant in opportunistic pathogens.^26^ The researchers showed that compared with the gut microbiomes of healthy persons, those of patients with COVID-19 had low abundances of the anti-inflammatory bacteria *Lachnospiraceae, Roseburia*, *Eubacterium*, and *Faecalibacterium prausnitzii*. The feces of COVID-19 patients were enriched in opportunistic pathogens known to cause bacteremia such as *Clostridium hathewayi*, *Enterobacteriaceae, Enterococcus*, *Actinomyces viscosus*, and *Bacteroides nordii*. Gut dysbiosis persists even after clearance of SARS-CoV-2 infection or recovery from it. The gut fungi and virome comprise parts of the gut microbiota and are also altered in response to SARS-CoV-2 infection. Another study observed relatively increased proportions of opportunistic fungal pathogens such as *Candida albicans*, *C. auris*, and *Aspergillus flavus* in the feces of COVID-19 patients.^25^ Previous investigations showed that the foregoing fungal pathogens are associated with pneumonia and other respiratory infections.^83–85^ Therefore, gut fungi dysbiosis might contribute to fungal co-infections and/or secondary fungal infection in COVID-19 patients. *Aspergillus* co-infection was recently isolated from the respiratory tract secretions and tracheal aspirates of COVID-19 patients.^86–90^

The gut virome helps regulate intestinal immune homeostasis.^91–93^ A recent study used in-depth shotgun sequencing to investigate relative changes in the fecal virome of COVID-19 patients.^27^ There were increased proportions (11/19) of eukaryotic DNA viruses and decreased proportions (18/26) of prokaryotic DNA viruses (and especially bacteriophages) in the feces of COVID-19 patients possibly because of SARS-CoV-2 infection. The abundance of fecal eukaryotic viruses may increase to take advantage of the host immune dysfunction that may occur in response to SARS-CoV-2 infection. Analysis of the modifications in gut virome functionality revealed that in COVID-19 patients, stress, inflammation, and virulence responses were comparatively increased and included arginine repressor, hemolysin channel protein, and DNA polymerase IV expression and DNA repair.

The foregoing studies helped elucidate the relationships between microbiota and SARS-CoV2 infection and could, therefore, disclose possible gut microbiota interventions that reduce disease severity in hospitalized COVID-19 patients.

### Gut dysbiosis is associated with post-acute COVID-19 syndrome (PACS)

Post-acute COVID-19 syndrome (PACS) is characterized by long-term complications and/or persistent symptoms following initial disease onset.^94–99^ The symptoms of PACS may be respiratory (cough, expectoration, nasal congestion/runny nose, and shortness of breath), neuropsychiatric (headache, dizziness, loss of taste, anosmia, anxiety, difficulty concentrating, insomnia, depression, poor memory, and blurred vision), gastrointestinal (nausea, diarrhea, and abdominal and epigastric pain), dermal (hair loss), musculoskeletal (arthralgia and muscle pain) and may also include fatigue.^96,100–109^ The underlying reasons for the emergence of PACS are unclear. A recent study revealed that gut dysbiosis might play a vital role in PACS.^110^ Stool samples were collected from 68 COVID-19 patients of whom 50 (73.5%) presented with PACS at six months after the initial COVID-19 diagnosis. There was no significant correlation between fecal or respiratory viral load and PACS development. However, a six-month follow-up indicated differences in the gut microbiota between patients with PACS and those without it. The gut microbiota of patients without PACS were comparable to those of healthy controls whereas those of patients with PACS substantially differed from those of the healthy controls at six months. In addition, patients with PACS have reduced bacterial diversity and richness than the healthy individuals. In contrast, the foregoing parameters did not significantly differ between patients without PACS and healthy controls. In the PACS patients, 28 and 14 gut bacterial species had decreased and increased, respectively, compared with the healthy controls. The authors examined the associations between the gut microbiome composition and the various PACS symptoms at six months. The R package MaAsLin2 (https://github.com/biobakery/Maaslin2) revealed that different PACS symptoms were related to different gut microbiota patterns. Eighty-one bacteria were associated with various PACS classes and many of these taxa were associated with at least two persistent symptoms.

The authors also investigated whether the gut microbiota profile at admission can influence PACS development. Analyses of stool samples at admission disclosed that bacterial clusters distinctly differed between patients with and without PACS. Compared with the PACS patients, those without PACS-COVID-19 presented with gut bacterial compositions that were enriched for 19 bacteria and characterized by *Bifidobacterium*, *Brautia*, and *Bacteroidetes*. Patients with PACS displayed significantly lower gut bacterial diversity and richness than those of healthy controls. Thirteen bacterial species including *Blautia wexlerae* and *Bifidobacterium longum* were negatively associated with PACS at six months. Hence, these species may have protective roles during recovery from SARS-CoV-2 infection. In contrast, *Actinomyces sp S6 Spd3*, *Actinomyces johnsonii*, and *Atopobium parvulum* were positively correlated with PACS. The authors also reported that certain bacterial species such as *Ruminococcus gnavus, Clostridium innocuum*, and *Erysipelatoclostridium ramosum* remained variable from admission to the 6-month follow-up and were associated with several PACS symptoms.

Taken together, the foregoing findings suggest that gut microbiota composition upon patient admission may reflect the susceptibility of the individual to long-term COVID-19 complications. As millions of people have been infected during the ongoing COVID-19 pandemic, the discoveries of the preceding studies strongly suggest that gut microbiota modulation could facilitate timely recovery from COVID-19 and reduce the risk of acute PACS development.

## Potential roles of microbiota in COVID-19

### Microbiota may contribute to cytokine storms in COVID-19 patients

Inflammation is a protective immune response that helps clear sources of infection. However, chronic or excessive inflammation can cause autoimmune damage.^111,112^ In the early stages of the pandemic, inflammatory cytokine storms were observed in certain COVID-19 patients.^113–115^ Cytokine storms are also known as inflammatory factor storms or systemic inflammatory response syndrome (SIRS).^116^ Excessive immunocyte activation releases large numbers of intracellular inflammatory factors including IL-6, IL-1β, TNF-α, IFN, and complement protein. Consequently, immunocytes mount storm-like suicide attacks on pathogens and infected cells, cause collateral damage to healthy cells and tissues, increase vascular permeability, and disturb circulation.^117^ The underlying mechanisms of the inflammatory factor storms induced by SARS-CoV-2 infection are assigned to the following three categories.

1. SARS-CoV-2 invades epidermal cells by binding the cell-surface receptors ACE2 and TMPRSS2, hijacks host cells, and undergoes self-replication. After large numbers of viruses are produced and released from the epithelial cells, innate lymphocytes such as macrophages and dendritic cells (DC) recognize and bind viral pathogen-associated molecular patterns (PAMPs) via pattern recognition receptors (PRRs) such as Toll-like receptors (TLRs), RIG-I-like receptors (RLRs), and NOD-like receptors (NLRs). These PRRs induce the expression of proinflammatory factors, IFNs, and numerous IFN-stimulated genes (ISGs) (Fig. 3).^118,119^

**Fig. 3:**
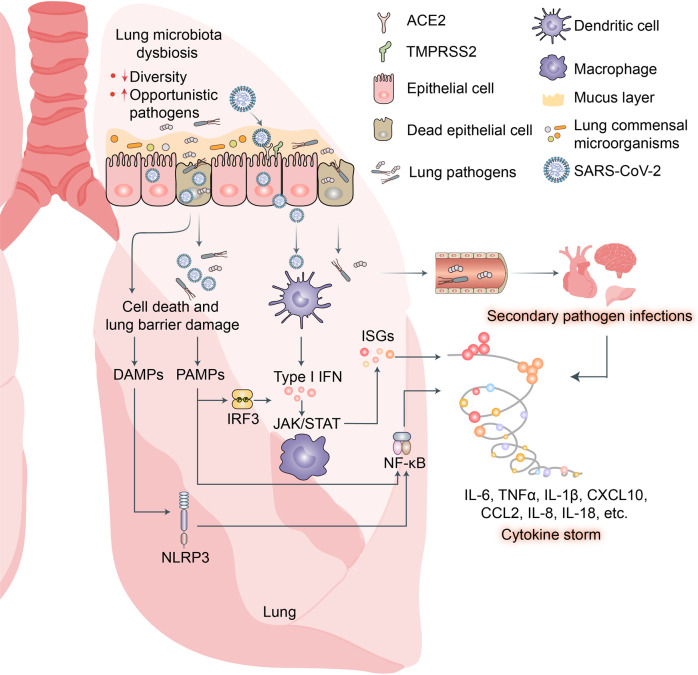
Potential mechanisms of cytokine storm and secondary pathogen infections resulting from lung microbiota dysbiosis in patients with COVID-19. SARS-CoV-2 infection disrupts lung microbiota eubiosis. Increased abundance of opportunistic pathogens may intensify lung cytokine storm and cause secondary pathogen infections in patients with COVID-19. Pathogen-associated molecular patterns (PAMPs) released from invading opportunistic pathogens may be recognized by host innate lymphocytes such as macrophages and dendritic cells (DCs) via pattern recognition receptors (PRR) including Toll-like receptors (TLRs), RIG-I-like receptors (RLRs), and NOD-like receptors (NLRs). These induce expression of proinflammatory factors via NF-κB signaling, interferons via IRF3 signaling, and numerous interferon-stimulated genes (ISGs) via JAK/STAT signaling. Excess cytokines may exacerbate COVID-19 symptoms
2. Cells killed by SARS-CoV-2 infection release multiple danger-associated molecular patterns (DAMPs) that activate the RLRs and NLRs and, by extension, promote the expression of various proinflammatory factors.^118,120^
3. SARS-CoV-2 infection disrupts respiratory and gastrointestinal microbiota eubiosis by decreasing the proportions of probiotics and increasing the abundance of opportunistic pathogens. It damages the respiratory and gastrointestinal epithelial cell mucosal layers.^121,122^ It also destroys the tight junctions (TJs) between epidermal cells (Fig. 4).^123^ These vital physical barriers prevent opportunistic pathogen invasion.^124–127^ In their absence, opportunistic pathogens may enter circulation and cause systemic inflammation and infection. The sodium-dependent neutral amino acid transporter B^0^AT1 or SLC6A19 may also be implicated in the disruption of the foregoing physical barriers and/or homeostasis by SARS-CoV-2 infection.^128^ B^0^AT1 is also a molecular ACE2 chaperone.^129^ ACE2 was required for B^0^AT1 expression on the luminal surfaces of murine intestinal epithelial cells.^130,131^ B^0^AT1 mediates neutral amino acid uptake by the luminal surfaces of intestinal epithelial cells.^132^ B^0^AT1 substrates such as tryptophan and glutamine activate the release of antimicrobial peptides, promote TJ formation, downregulate lymphoid proinflammatory cytokines, and modulate mucosal cell autophagy via mTOR signaling.^133,134^ As ACE2 is a molecular B^0^AT1 chaperone, both molecules may be co-internalized during SARS-CoV-2 infection and the net amount of B^0^AT1 on the cell membrane surface may decrease. A recent study corroborated this hypothesis.^135^ Cryoelectron microscopy was used to examine the ultrastructures of the ACE2-B^0^AT1 complex as well as another one involving the SARS-CoV-2 receptor-binding domain (RBD). The analysis disclosed that the ACE2-B^0^AT1 complex exists as a heterodimer and the SARS-CoV-2 spike protein (S1) may interact with it.^135^ SARS-CoV-2-induced B^0^AT1 downregulation on the luminal surfaces of intestinal epithelial cells might contribute to microbiota dysbiosis which, in turn, promotes pathogen invasion and ultimately facilitates cytokine storms and COVID-19 exacerbation.^134^

**Fig. 4:**
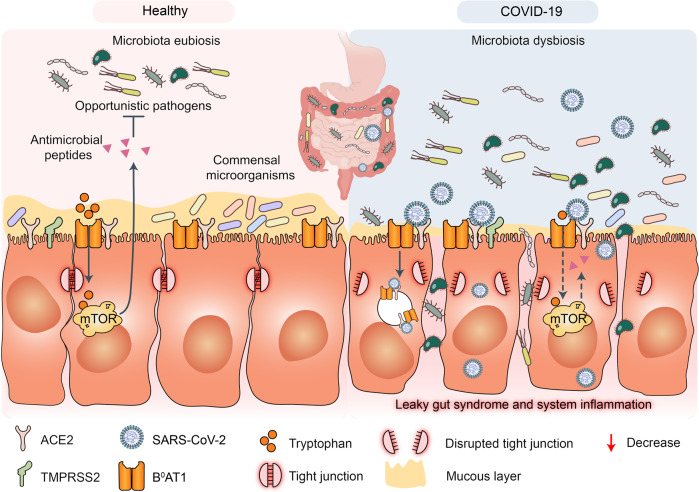
Potential mechanisms of cytokine storm and secondary pathogen infections resulting from gut microbiota dysbiosis in patients with COVID-19. Gut microbiota are also disrupted by SARS-CoV-2 infection which potentially triggers cytokine storm and secondary pathogen infections. B^0^AT1 mediates neutral amino acid uptake by luminal surfaces of intestinal epithelial cells. It is also a molecular ACE2 chaperone. B^0^AT1 substrates such as tryptophan and glutamine activate antimicrobial peptide release, promote tight junction (TJ) formation, downregulate lymphoid proinflammatory cytokines, and modulate mucosal cell autophagy via mTOR signaling. As ACE2 is a molecular B^0^AT1 chaperone, ACE2-associated B^0^AT1 may be internalized during SARS-CoV-2 infection, decrease B^0^AT1 on cell membranes, promote gut opportunistic pathogen invasion, facilitate cytokine storms, and exacerbate COVID-19

### Gut commensal-derived metabolites and components modulate lung antiviral immune responses via the gut–lung axis

Gut microbiota metabolites are small molecules produced as intermediate or end products of gut microbial metabolism. They are derived either from the bacterial metabolism of dietary substrates or directly from the bacteria themselves.^136^ Gut microbiota-derived metabolites are the main mediators of gut microbiota-host interactions that influence host immunity. Hence, we will discuss the potential mechanisms by which gut microbiota-derived metabolites modulate the host immune responses to SARS-CoV-2 infection.

#### Host defense in the early stages of SARS-CoV-2 infection

**Fig. 5:**
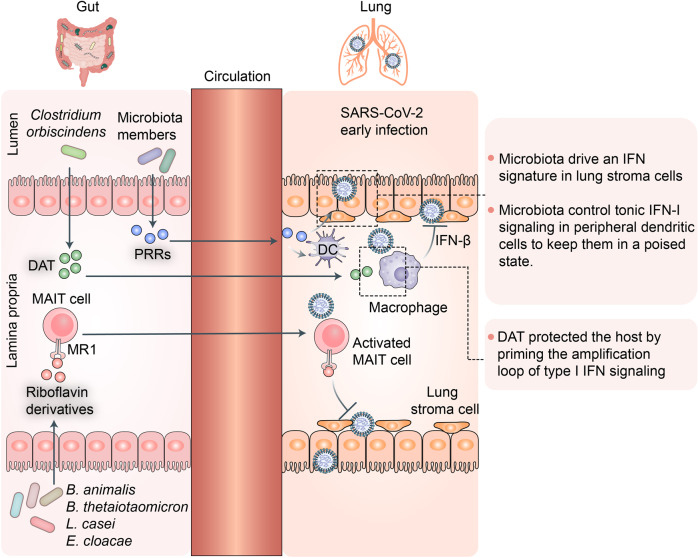
Gut commensal-derived metabolites or components potentially promote lung antiviral immune responses via gut–lung axis during the early stages of SARS-CoV-2 infection. Gut microbiota such as *Bifidobacterium animalis, Bacteroides thetaiotaomicron, Lactobacillus casei*, and *Enterobacter cloacae* generate riboflavin derivatives that activate mucosal-associated T cells (MAIT) via restrictive major histocompatibility complex (MHC)-related protein-1 (MR1)-mediated recognition. Activated gut MAIT cells may participate in lung antiviral immune responses via gut–lung axis during early stages of SARS-CoV-2 infection. Deaminotyrosine (DAT) generated by gut bacterium *Clostridium orbiscindens* may protect host from viral infection by initiating amplification loop of type I interferon (IFN) signaling. Microbiota-derived components are required to program DCs during steady state so they can rapidly initiate immune responses to pathogens. Gut microbiota-derived metabolites and components may play vital roles in inhibiting early SARS-CoV-2 infection

Mucosal-associated T cells (MAIT) constitute an evolutionarily conserved T-cell subset with innate functions resembling those of innate natural killer T cells (iNKT) cells.^137^ They are localized mainly to the spleen, lymph nodes, and liver. Nevertheless, they may also inhabit barrier tissues such as the lung, skin, and gut.^138^ They respond to pathogens via restrictive major histocompatibility complex (MHC)-related protein-1 (MR1)-mediated recognition of riboflavin derivatives produced by gut microbiota such as *Bifidobacterium animalis, Bacteroides thetaiotaomicron, Lactobacillus casei*, and *Enterobacter cloacae.*^139,140^ These microbially-derived signals affect all stages of MAIT cell biology including intrathymic development, peripheral expansion, and organ function.^141,142^ In tissues, MAIT cells integrate multiple signals and display effector functions associated with defense against infectious pathogens.^143^ A recent study showed that MAIT cells are highly involved in the host immune response against COVID-19.^144^ MAIT cells participate in both local and systemic immune responses in the airways during the early stages of SARS-CoV-2 infection. They are recruited by proinflammatory signals from the blood into the airways and rapidly promote an innate immune response against SARS-CoV-2 infection (Fig. 5).

Deaminotyrosine (DAT) is a bacterial metabolite derived from flavonoids. It was recently demonstrated that DAT protects the host from influenza infection by initiating a type I interferon (IFN) signaling amplification loop.^145^ The authors used a reporter cell line harboring multiple type I IFN response elements to screen a library of 84 microbe-associated metabolites and found that DAT significantly affected IFN signaling. Mice administered DAT after influenza infection exhibited reduced mortality, lower viral gene expression, and decreased proportions of apoptotic cells in their airways. The authors analyzed changes in fecal and serum DAT content in antibiotic-treated mice and confirmed that their gut microbiota produced this compound. The researchers also reported that the human gut bacterium *Clostridium orbiscindens* degrades flavonoids to DAT. Gut microbiota-derived components such as lipopolysaccharides (LPS) also help protect lungs from viral infections.^146^ Recent evidence from Schaupp et al. suggested that microbiota-derived components are required to program dendritic cells (DCs) in steady-state so that they rapidly respond to pathogens and initiate immune responses against them.^147^ Another study showed that gut microbiota-driven tonic IFN signals in lung stromal cells protect the host against influenza virus infection.^148^ SARS-CoV-2 and influenza virus are similar in many ways. Thus, gut microbiota-derived metabolites and components might help inhibit early SARS-CoV-2 infection.^149^

#### Anti-inflammation

**Fig. 6:**
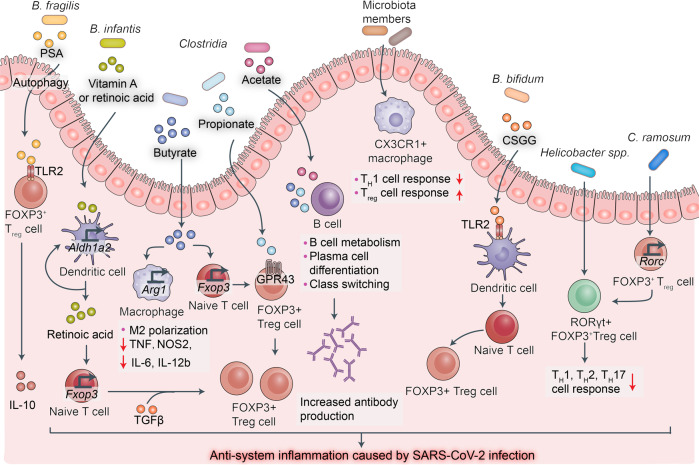
Anti-inflammatory immunomodulation by gut microbiota-derived metabolites and components. Left to right: polysaccharide A (PSA) capsular component of gut commensal *Bacteroides fragilis* can be transported to gut lamina propria via autophagy-related protein 16-like 1 (ATG16L1) and nucleotide-binding oligomerization domain-containing protein 2 (NOD2)-dependent autophagy. PSA signals promote FOXP3 + regulatory T-cell (Treg) proliferation and IL-10 production and induce an anti-inflammatory state. Vitamin A or retinoic acid (RA) derived from gut commensal *Bifidobacterium infantis* upregulates *Aldh1a2* encoding retinal dehydrogenase 2 in DCs. *Aldh1a2*-expressing DCs produce high levels of RA which collaborates with transforming growth factor-β (TGF-β) to promote naive T cell differentiation into FOXP3 + Treg cells and inhibit inflammation caused by SARS-CoV-2 infection. Gut microbiota may produce short-chain fatty acids (SCFAs) acetate, propionate, and butyrate to inhibit inflammation caused by SARS-CoV-2 infection. Butyrate promotes M2-like macrophage polarization and anti-inflammatory activity by upregulating arginase 1 (ARG1), suppressing tumor necrosis factor (TNF) production, and downregulating *Nos2, Il6*, and *Il12b*. Butyrate inhibits histone deacetylases, increases transcription at Foxp3 promoter and related enhancer sites in naive T cells, and promotes naive T cell differentiation into Treg cells. Propionate activates GPR43 on Treg cells, thereby enhancing their proliferation. Acetate promotes anti-SARS-CoV-2 antibody production in B cells, thereby inhibiting SARS-CoV-2 infection. Microbiota members induce CX3CR1 + macrophages that inhibit T helper 1 (TH1) and promote Treg cell responses. Cell-surface β-glucan/galactan (CSGG) polysaccharide produced by *Bifidobacterium bifidum* may induce Foxp3+ regulatory T-cell generation and inhibit inflammation caused by SARS-CoV-2 infection. Gut commensals such as *Helicobacter* spp. and *Clostridium ramosum* induce RORγt expression in Foxp3+ regulatory T cells, thereby suppressing TH1, TH2, and TH17 cell-type inflammatory responses

Proinflammatory cytokine storms caused by SARS-CoV-2 infection are associated with severe disease and high mortality rates. Several drugs suppressing or attenuating proinflammatory cytokine storms have been administered in the clinical treatment of severe or critical COVID-19 patients.^150,151^ Siltuximab is a monoclonal antibody targeting IL-6R.^152^ Numerous studies showed that various microbial metabolites inhibit inflammation. Therefore, in this section, we will discuss the putative mechanisms by which these substances suppress COVID-19-related inflammation (Fig. 6).

Short-chain fatty acids (SCFAs) are produced by various bacterial groups. They include acetate (50–70%; formed by many bacterial taxa), propionate (10–20%; synthesized by *Bacteroidetes* and certain *Firmicutes*), and butyrate (10–40%; generated by a few Clostridia).^153^ SCFAs influence immune responses in the gut and those associated with peripheral circulation and distal body sites^154–158^ A recent study by Kim et al. found that the SCFAs produced by microbiota enhanced B cell metabolism and gene expression and supported optimal homeostatic and pathogen-specific antibody responses.^159^ SCFAs have these effects on the B cells in the gut and systemic tissues. Therefore, SCFAs derived from gut microbiota may promote anti-SARS-CoV-2 antibody production in B cells and inhibit COVID-19 development. Another study revealed that the gut microbiome of patients with COVID-19 presented with impaired SCFA capacity even after disease resolution.^160,161^ Thus, there may be a direct link between the severity of COVID-19 infection and persistent impairment of gut microbiota metabolism.

SCFAs also inhibit inflammation by modulating various immunocytes. Butyrate promotes M2-like macrophage polarization and, by extension, anti-inflammatory activity by upregulating arginase 1 (ARG1) and ultimately downregulating TNF, Nos2, IL-6, and IL-12b.^162^ Regulatory T cells (Treg cells) comprise a T-cell subset with significant immunosuppressive effects and the capacity to express Foxp3, CD25, and CD4. A variety of anti-inflammatory cytokines secreted from Treg cells can inhibit auto-inflammatory responses, and prevent pathological immune responses from causing tissue damage.^163^ Defective or absent Treg cell function may result in inflammatory disease.^164–166^ Butyrate can promote the differentiation of naive T cells into Treg cells by inhibiting histone deacetylase or increasing the transcription of Foxp3 promoter in naive T cells.^155,157^ Propionate activates GPR43 on Treg cells and enhances their proliferation.^167^ Other microbiota-derived metabolites and components also modulate Treg cells.

*Bifidobacterium infantis-*derived vitamin A or retinoic acid (RA) upregulates *Aldh1a2* encoding retinal dehydrogenase 2 in DCs.^168–170^ *Aldh1a2*-expressing DCs produce high levels of RA and this substance collaborates with transforming growth factor-β (TGF-β) to promote naive T cell differentiation into FOXP3^+^ Treg cells.^168,169,171^ The capsular component polysaccharide A (PSA) of the gut commensal *Bacteroides fragilis* can be transported to the gut lamina propria via autophagy-related protein 16-like 1 (ATG16L1) and the nucleotide-binding oligomerization domain-containing protein 2 (NOD2)-dependent autophagy pathway.^172–174^ Toll-like receptor 2 (TLR2) on FOXP3^+^ Treg cells recognize PSA signals which, in turn, induce FOXP3^+^ Treg cell proliferation, IL-10 production, and an anti-inflammatory state.^175–177^ Cell-surface β-glucan/galactan (CSGG) polysaccharide produced by *Bifidobacterium bifidum* promotes Foxp3^+^ Treg cell generation.^178^ Retinoic acid receptor-related orphan receptor gamma t (RORγt; a nuclear hormone receptor) may induce proinflammatory T helper 17 (T_H_17) cell differentiation.^179^ Recent studies showed that certain gut commensals such as *Helicobacter* spp. and *Clostridium ramosum* can induce RORγt expression in Foxp3^+^ Treg cells.^180,181^ RORγt^+^ Foxp3^+^ Treg cells downregulate T_H_1-, T_H_2-, and T_H_17 cell-type immune responses.^180–182^

Various gut commensal-derived metabolites and components such as vitamins, carbohydrates, amino acid derivatives, and glycolipids have been proved to modulate multiple host immunocyte subsets by different mechanisms. The immunomodulatory effects of gut commensals are potential targets for the design and administration of SARS-CoV-2 prophylaxes and therapies.

## Targeting microbiota as an auxiliary for COVID-19 prevention and treatment

**Fig. 7:**
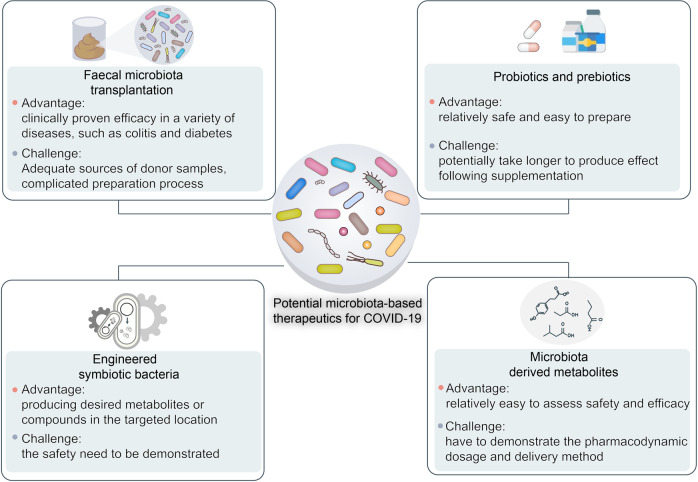
Potential microbiota-based COVID-19 therapies include fecal microbiota transplantation (FMT), probiotics and prebiotics, engineered symbiotic bacteria, and microbiota-derived metabolites

Gut microbiota are vital to immunomodulation. Thus, microbiota-based therapies such as fecal microbiota transplantation (FMT), probiotics, and prebiotics have been used in the clinical treatment of various human diseases such as diabetes, obesity, cancers, ulcerative colitis, Crohn’s disease, and certain viral infections.^183^ Recent studies have manipulated gut microbiota to treat COVID-19 and its complications.^184–190^ Here, we review and discuss putative microbiota-based COVID-19 therapies (Fig. 7).

In FMT, feces or complex microbial communities derived from in vitro culture or purification of fecal material from a healthy donor are inoculated into the intestinal tract of a patient. FMT has demonstrated efficacy against colitis, diabetes, and recurrent *Clostridioides difficile* infection.^191–194^ Recently, a registered clinical trial (ClinicalTrials.gov Identifier No. NCT04824222) attempted to validate the efficacy of FMT as an immunomodulatory risk reducer in COVID-19 disease progression associated with escalating cytokine storms and inflammation. The control group is administered standard pharmacological treatments while the experimental group is also orally administered FMT in the form of 30–50 dose-in, double-cover, gastro-resistant, enteric-release frozen 60-g capsules.^195^ A main outcome measure is the incidence of adverse events in the safety pilot group up to day 30 after administration. Another outcome metric is the percentage of patients in the study and control groups requiring escalation of non-invasive oxygen therapy modalities such as increasing FiO2, administering high-flow nasal cannula oxygen therapy (HFNOT), continuous positive airway pressure (CPAP), or invasive ventilation, ventilators, and/or ICU hospitalization corresponding to grades 5–7 disease exacerbation on the COVID-19 performance status scale. This trial is still in progress. Nevertheless, considering the vital roles of gut microbiota in immune regulation, we believe that FMT is a possible therapeutic option for suppressing COVID-19-induced cytokine storms and inflammation.

Supplementation with microbiota-targeted substrates (prebiotics) such as specific dietary fibers and/or direct transfer of one or several specific beneficial microbiota (probiotics) are promising COVID-19 treatment approaches that modulate the gut microbiota.^186,196^ Treatment with probiotics and/or prebiotics is relatively safer and easier to prepare and administer than FMT. The National Health Commission of China has recommended the clinical administration of probiotics to patients with severe COVID-19 for the purposes of restoring and maintaining gut microflora balance and preventing secondary infection. Indeed, numerous clinical trials are validating the efficacy of probiotics and/or prebiotics at reducing COVID-19 duration and symptoms (Table 1). One clinical trial (ClinicalTrials.gov Identifier No. NCT05043376) is investigating the efficacy of the probiotic *Streptococcus salivarius* K12 (BLIS K12)^197^ in hospitalized COVID-19 patients. Investigators in a phase II randomized clinical trial (ClinicalTrials.gov Identifier No. NCT05175833) are assessing the efficacy of BLIS K12 and *Lactobacillus brevis* CD2 in the prevention of secondary bacterial pneumonia in patients with severe COVID-19. Another randomized trial (ClinicalTrials.gov Identifier No. NCT04399252) at Duke University Hospital is evaluating the efficacy of the probiotic *Lactobacillus rhamnosus* GG at preventing COVID-19 transmission and symptom development in exposed household contacts. None of the foregoing clinical trials has yet published the results. However, it has already been empirically demonstrated that certain probiotic stains have antiviral activity against other coronaviruses. Therefore, probiotics could potentially be used in the prevention and/or adjuvant treatment of COVID-19.

**Table 1:** Examples of completed clinical trials evaluating the efficacy of probiotics or prebiotics in the treatment of COVID-19

Title	Interventions	Population	Locations	Clinical trial ID
Efficacy of Probiotics in Reducing Duration and Symptoms of COVID-19	• Dietary supplement: probiotics (2 strains 10 × 10^9^ UFC) • Dietary supplement: placebo (potato starch and magnesium stearate)	Enrollment: 17	• CIUSSS de L’Estrie-CHUS Hospital, Sherbrooke, Quebec, Canada	NCT04621071
Study to Evaluate the Effect of a Probiotic in COVID-19	• Dietary supplement: probiotic • Dietary supplement: placebo	Enrollment: 41	• Hospital Universitario del Vinalopó, Elche, Alicante, Spain • Hospital Universitario de Torrevieja, Torrevieja, Alicante, Spain	NCT04390477
Efficacy of Intranasal Probiotic Treatment to Reduce Severity of Symptoms in COVID-19 Infection	• Dietary supplement: probiotic • Dietary supplement: saline solution	Enrollment: 23	• Centre Hospitalier de l’Université de Montréal (CHUM), Montreal, Quebec, Canada	NCT04458519
The Effect of Probiotic Supplementation on SARS-CoV-2 Antibody Response After COVID-19	• Dietary supplement: *L. reuteri* DSM 17938 + vitamin D • Dietary supplement: placebo + vitamin D	Enrollment: 161	• Örebro University, Örebro, Örebro Län, Sweden	NCT04734886
Study to Investigate the Treatment Benefits of Probiotic Streptococcus Salivarius K12 for Hospitalized Patients (Non-ICU) With COVID-19	• Drug: standard of care • Dietary supplement: BLIS K12	Enrollment: 50	• King Edward Medical University Teaching Hospital, Lahore, Punjab, Pakistan	NCT05043376
Oral Probiotics and Secondary Bacterial Pneumonia in Severe COVID-19	• Combination product: oral probiotics • Other: oral placebo	Enrollment: 70	• University of Passo Fundo, Passo Fundo, RS, Brazil	NCT05175833
Live Microbes to Boost Anti-Severe Acute Respiratory Syndrome Coronavirus-2 (SARS-CoV-2) Immunity Clinical Trial	• Dietary supplement: OL-1, standard dose • Dietary supplement: OL-1, high dose • Dietary supplement: placebo	Enrollment: 54	• Rutgers University, New Brunswick, New Jersey, United States	NCT04847349
Efficacy of Probiotics in the Treatment of Hospitalized Patients With Novel Coronavirus Infection	• Dietary supplement: probiotic • Dietary supplement: placebo	Enrollment: 200	• I.M. Sechenov First Moscow State Medical University, Moscow, Russian Federation	NCT04854941
Efficacy of *L. plantarum* and *P. acidilactici* in Adults With SARS-CoV-2 and COVID-19	• Dietary supplement: probiotic • Dietary supplement: placebo	Enrollment: 300	• Hospital General Dr. Manuel Gea Gonzalez, Mexico city, Mexico	NCT04517422
Effect of Lactobacillus on the Microbiome of Household Contacts Exposed to COVID-19	• Dietary supplement: *Lactobacillus rhamnosus* GG • Dietary Supplement: *Lactobacillus rhamnosus* GG placebo	Enrollment: 182	• Duke University, Durham, North Carolina, United States	NCT04399252
Effect of a NSS to Reduce Complications in Patients With Covid-19 and Comorbidities in Stage III	• Dietary supplement: nutritional support system (NSS) • Other: conventional nutritional support designed by hospital nutritionists	Enrollment: 80	• ISSEMYM “Arturo Montiel Rojas” Medical Center, Toluca de Lerdo, Mexico State, Mexico	NCT04507867

All of the data from https://clinicaltrials.gov/

Advances in synthetic biology and gene manipulation are facilitating and realizing the design of microorganisms based on therapeutic requirements for COVID-19. We can now engineer symbiotic bacteria with desired functions, the ability to produce the required metabolites, and the capacity to target the correct locations in the host. A *Lactococcus lactis* strain was engineered to express and secrete the anti-inflammatory cytokine IL-10 to treat colitis.^198^ The biosafety of this strain was ensured by making it require exogenous thymidine for survival and IL-10 production.^199^ The cytokine storms caused by SARS-CoV-2 infection have a close relationship with COVID-19 severity and mortality. Hence, the design and application of similarly engineered strains to produce anti-inflammatory metabolites in the lungs and suppress proinflammatory storms could culminate in a promising COVID-19 treatment. While much further clinical study is required to validate the safety and efficacy of this technology. Direct supplementation of beneficial microbiota-derived metabolites such as SCFAs are also promising candidates for COVID-19 treatment.

Emerging evidence from interventional studies and animal models suggests that the microbiota plays a crucial role in antibody responses to vaccination.^200–205^ For example, antibiotic-treated and germ-free mice had reduced antibody responses to the seasonal influenza vaccine.^206^ Therefore, in addition to the COVID-19 treatment, considering microbiota as a vital factor modulating immune responses to vaccination, microbiota-targeted interventions are a promising way to optimize the COVID-19 vaccine effectiveness. However, so for, relatively few studies have evaluated the effects of the microbiota on immune responses to COVID-19 vaccination and further work is required in this area.

## Conclusions and perspectives

Symptoms associated with the initial phase of COVID-19 include dry cough, shortness of breath, vomiting, and diarrhea.^15,20,207^ The respiratory and gastrointestinal tracts are the primary habitats of human microbiota and targets for SARS-CoV-2 infection as they express ACE2 and TMPRSS2 at high levels.^44,46,208–212^ There is growing evidence that the substantial perturbation of these microbiota during COVID-19 is associated with disease severity and mortality and post-acute COVID-19 syndrome (PACS).^26–28,110,160,213^ Microbiota are powerful immunomodulatory factors in human health and disease.^214–217^ Hence, targeting microbiota manipulation is a promising strategy for the prevention and treatment of COVID-19 and PACS. Numerous clinical trials are evaluating the efficacy of adjuvant therapy with probiotics as well as other microbiota-based treatments. However, the outcomes of these clinical trials have not yet been published. Additional clinical data are required to validate the safety and efficacy of microbiota-based therapies for patients with COVID-19 or PACS.

The SARS-CoV-2 omicron variant has recently and rapidly spread worldwide.^218^ Notably, the Omicron variant is not a single strain, but evolved into three lineages: BA.1, BA.2, and BA.3.^219^ BA.1 was once the most widely prevalent strain in the world; however, BA.2 is suggested to be more transmissible than the BA.1 and BA.2 is gradually replacing BA.1 in several countries, such as Denmark, Nepal, and the Philippines.^220^ The transmissibility of BA.3 is very limited, with very few cases, at most a few hundred cases. Certain studies proposed that the omicron variant can evade infection- and vaccination-induced antibodies and exacerbate existing public health risks.^221–227^ In contrast, other studies demonstrated comparatively lower hospitalization rates associated with the omicron variant than the wild type SARS-CoV-2.^228–230^ However, the differences among the omicron and wild type strains in terms of their relative impact on host microbiota alterations are unknown. Future investigations might help develop microbiota-based therapeutics customized for omicron variant infections.

Not only various intrinsic host factors (such as age, sex, genetics, and comorbidities), but also extrinsic factors (such as rural versus urban location, geographical location, season, and toxins) have been shown to influence the composition of the microbiota.^231^ Moreover, microbiota composition varies widely among individuals and populations.^232^ They also greatly differ in terms of their SARS-CoV-2 symptoms. Cases may range from asymptomatic to acute pneumonia.^17^ However, there is little data available on the associations among microbiota composition and coronavirus susceptibility. Thus, clarification of the relationships between SARS-CoV-2 susceptibility and microbiota composition may facilitate the design and deployment of prophylactic and therapeutic measures against the new SARS-CoV-2 strains. It is clear that microbiota are strongly implicated in host immune responses to various diseases including COVID-19. Nevertheless, it remains to be determined whether microbiota-based therapeutics influence COVID-19 outcome. This research focus should be prioritized as the COVID-19 pandemic continues to be severe in certain parts of the world.
